# Multi-trait, Multi-environment Deep Learning Modeling for Genomic-Enabled Prediction of Plant Traits

**DOI:** 10.1534/g3.118.200728

**Published:** 2018-10-04

**Authors:** Osval A. Montesinos-López, Abelardo Montesinos-López, José Crossa, Daniel Gianola, Carlos M. Hernández-Suárez, Javier Martín-Vallejo

**Affiliations:** * Facultad de Telemática; ** Facultad de Ciencias, Universidad de Colima, Colima, Colima, 28040, México; † Departamento de Matemáticas, Centro Universitario de Ciencias Exactas e Ingenierías (CUCEI), Universidad de Guadalajara, 44430, Guadalajara, Jalisco, México; ‡ Biometrics and Statistics Unit, Genetic Resources Program, International Maize and Wheat Improvement Center (CIMMYT), Apdo. Postal 6-641, 06600, Ciudad de México, México; § Departments of Animal Sciences, Dairy Science, and Biostatistics and Medical Informatics, University of Wisconsin-Madison, Madison, Wisconsin 53706; †† Departamento de Estadística, Universidad de Salamanca, c/Espejo 2, Salamanca, 37007, España

**Keywords:** deep learning, multi-trait, multi-environment, genomic prediction, plant breeding, Bayesian modeling, GenPred, Shared Data Resources

## Abstract

Multi-trait and multi-environment data are common in animal and plant breeding programs. However, what is lacking are more powerful statistical models that can exploit the correlation between traits to improve prediction accuracy in the context of genomic selection (GS). Multi-trait models are more complex than univariate models and usually require more computational resources, but they are preferred because they can exploit the correlation between traits, which many times helps improve prediction accuracy. For this reason, in this paper we explore the power of multi-trait deep learning (MTDL) models in terms of prediction accuracy. The prediction performance of MTDL models was compared to the performance of the Bayesian multi-trait and multi-environment (BMTME) model proposed by Montesinos-López *et al.* (2016), which is a multi-trait version of the genomic best linear unbiased prediction (GBLUP) univariate model. Both models were evaluated with predictors with and without the genotype×environment interaction term. The prediction performance of both models was evaluated in terms of Pearson’s correlation using cross-validation. We found that the best predictions in two of the three data sets were found under the BMTME model, but in general the predictions of both models, BTMTE and MTDL, were similar. Among models without the genotype×environment interaction, the MTDL model was the best, while among models with genotype×environment interaction, the BMTME model was superior. These results indicate that the MTDL model is very competitive for performing predictions in the context of GS, with the important practical advantage that it requires less computational resources than the BMTME model.

The key principle of genomic selection (GS) is to build an accurate prediction model based on a training population consisting of individuals with both genotypic and phenotypic data. Existing GS prediction models can be grouped into two main categories based on the number of traits analyzed: univariate-trait (UT) models and multi-trait (MT) models. Most of the time, UT models are trained to predict the value of a single continuous (or categorical) phenotype in a testing data set. When there are many traits (or variables), breeders need to use more complex analyses in order to obtain all the necessary information from the data (Everitt and Dunn 1992). For this reason, these situations are handled by a generalization of univariate models, which involves predicting multiple traits; the generalized models are known as MT models.

MT models are concerned with the simultaneous prediction of multiple traits based on the same set of explanatory input variables. It is assumed that MT data sets are generated by a single system, most likely indicating that the captured outputs have some structure. MT models are designed to more efficiently capturing the complex relationships between traits, and most of the time they produce more accurate parameter estimates and better predictions than the UT models. MT models exploit not only the correlation between lines, but also the correlation between traits, which improves its efficiency. UT models, on the other hand, eliminate any possibility of learning from the possible relationships between traits because a single, independent model is trained for each trait separately. Another advantage of MT techniques is model interpretability (Xiong *et al.*, 2014). A MT model is much more interpretable than a series of single-trait models because it not only exploits the relationship between lines, but also among the traits themselves. In addition, a MT deep learning model has the advantage that it does not increase the computational time exponentially when going from the univariate to the multivariate version, which makes MT deep learning very attractive.

MT models have recently become increasingly popular in GS due to their great capacity to predict multiple traits simultaneously and also because they help increase prediction accuracy when the traits are correlated. MT models are also popular in other fields like ecological modeling, energy forecasting, data mining (Wang *et al.*, 2015), computer vision (Yan *et al.*, 2015), water quality monitoring, forest monitoring, load/price forecasting and medical image analysis (Zhen *et al.*, 2015), due to their great effectiveness in solving challenging problems in a broad range of applications. Also, as one of the reviewers suggested, there is a great need for multivariate models in emerging fields like high-throughput phenotyping, where various traits are produced and not exploited to their full capacity due to the lack of adequate multivariate techniques that can exploit the genetic association among phenotypic traits. For dealing with multivariate data for high-throughput phenotyping and longitudinal interdependencies, we suggest reading the work of Sun *et al.* (2017), and for studying the relationship among traits with multivariate mixed models, we suggest reading Xavier *et al.* (2017).

In the context of GS, Jia and Jannink (2012) and Jiang *et al.* (2015) showed that the larger the genetic correlation between traits, the better the prediction accuracy of MT analysis compared to UT analysis. Calus and Veerkamp (2011) reported modest improvement in the prediction accuracy of MT analysis with regard to UT analysis. Montesinos-López *et al.* (2016) found modest improvement in the prediction accuracy of MT analysis for correlated traits in comparison to data with null correlation between traits. Along these lines, He *et al.* (2016) found that MT analysis improves prediction accuracy for correlated traits as compared to UT analysis. Schulthess *et al.* (2017) also found that in terms of prediction accuracy, MT analysis performs better than UT analysis, and pointed out that MT models are better when the degree of relatedness between genotypes is weaker. Also, there is evidence that MT analysis improves accuracy when classifying and identifying superior genetic constitutions (Montesinos-López *et al.*, 2018) and helps scientists understand the type of allele interaction involved in heredity and the relationships between the traits under study (Bertan *et al.*, 2009). In addition, it increases the precision of genetic correlation parameter estimates between traits, which helps breeders perform indirect selection. In general, MT analysis is a powerful tool for clarifying the relationship and the effect of each studied variable and for building more efficient prediction models (Castro *et al.*, 2013; Huang *et al.*, 2015).

It is documented that MT models have some advantages over UT models, including: (a) higher prediction accuracy for individual traits because there is more information (direct or indirect) and better data connectedness (Colleau *et al.*, 2009), especially when traits with varying heritabilities are analyzed jointly; this is true if genetic correlations are significant or substantial with low error correlations; (b) simplified index selection because optimal weight factors for the total merit index are the economic weights (Colleau *et al.*, 2009); and (c) procedures for obtaining genetic and residual covariances and incorporating these in expected breeding value (EBV) estimates for across-location, across-country or across-region evaluations (Schaeffer 2001).

Genomic prediction plays a significant role in the selection of the best candidate lines for which there is only measured genomic information. Achieving accurate phenotypic prediction using genetic information is a major goal in GS and plant breeding programs. Accurate prediction models will have great impact on selecting the best lines and on breeding program strategies. Various approaches for modeling MT data have been proposed in the context of GS. The most popular approach for MT prediction modeling in plant breeding is the use of mixed models under a frequentist and Bayesian approach. Selection by mixed models focusing on multivariate analyses is a powerful tool for selecting cultivars under the Bayesian approach of mixed models. One of these models is the Bayesian multi-trait and multi-environment (BMTME) model proposed by Montesinos-López *et al.* (2016), which is a MT version of the genomic best linear unbiased prediction (GBLUP) univariate model. Under a frequentist approach, the restricted maximum likelihood (Software ASREML; Gilmour *et al.*, 1995) is one of the most popular models in the context of mixed models.

Deep neural networks referring to artificial neural networks with more than two hidden layers, have been explored for prediction in many domains. Deep learning (DL) is often presented in the media as a field that appeared from nowhere during the last decade. However, the key concepts of DL have been developing for more than 60 years, since DL is a specific subfield of machine learning that deals with neural networks with more than two layers. The applications of DL cover many areas, for example, Qi *et al.* (2012) developed a unified multi-task, local-structure predictor of proteins using DL networks as a classifier. Fox *et al.* (2018) used DL models to accurately predict blood glucose trajectories. Spencer *et al.* (2015) developed an iterative DL network for protein secondary structure prediction. Tavanaei *et al.* (2017) used a DL model for predicting tumor suppressor genes and oncogenes. DL models have also made accurate predictions of single-cell DNA methylation states (Angermueller *et al.*, 2017). Alipanahi *et al.* (2015) used DL with a convolutional network architecture to predict specificities of DNA- and RNA-binding proteins. Menden *et al.* (2013) applied DL models to predict the viability of a cancer cell line exposed to a drug.

In a recent study, Montesinos-López *et al.* (2018) explored DL models with densely connected network architecture on nine extensive genomic data sets, including genomic×environment interaction, and compared the results to those of the GBLUP model. Results showed that the DL models appeared to be competitive, since they had higher prediction accuracy than the GBLUP in 6 out of the 9 data sets in scenarios that ignored the genotype×environment interaction term. However, the authors applied univariate modeling and did not attempt to add other traits to the prediction accuracy.

Based on the previous considerations and the need to adapt DL methodology to the application of GS in plant breeding, in this article we propose a MT deep learning (MTDL) model for genomic-enabled prediction of multiple response variables simultaneously. To evaluate its performance, we compare the MTDL model against the BMTME model, which is a Bayesian model for predicting multi-trait and multi-environment data in the context of GS (Montesinos-López *et al.*, 2016, 2018). Prediction performance was evaluated on three real data sets using 10 random cross-validations and measuring the prediction accuracy based on Pearson’s correlation between the observed and predicted values.

## Materials and Methods

### Implemented models

#### Bayesian multiple-trait multiple-environment (BMTME) model:

This model was implemented by Montesinos-López *et al.* (2016) and for a complete understanding of the description of the BMTME model, first we provide the notations used for the matrix-variate normal distribution that plays a key role in building the BMTME model. Matrix-variate normal distribution is a generalization of the multivariate normal distribution to matrices. The (*n*×*p*) random matrix, M, is distributed as matrix-variate normal distribution denoted as $M∼NM_{n\times p}\left(H,\Omega ,\Sigma \right)$, if and only if, the (*np*×1) random vector vec(M) is distributed as multivariate normal as $N_{np}\left(\text{vec}(H),\Sigma ⊗\Omega \right)$; therefore, $NM_{n\times p}$ denotes the (n×p) dimensional matrix-variate normal distribution, H is a (*n*×*p*) location matrix, Σ is a (p×p) first covariance matrix, and Ω is a (n×n) second covariance matrix (Srivastava and Khatri 1979). vec(.) and ⊗ are the standard vectorization operator and Kronecker product, respectively. Unlike in a multivariate normal model where the data are concatenated into a single vector of length *np*, in a matrix-variate normal model, the data (M) are in an *n*×*p* matrix where each column is a trait (Montesinos-López *et al.*, 2016). Therefore, the proposed BMTME model is defined as follows:

$$Y=\mathrm{X\beta}+Z_{1}b_{1}+Z_{2}b_{2}+E$$ (1)

where Y is of order n×L, with L the number of traits and n=J×I, where J denotes the number of lines and I the number of environments, X is of order n×I, β is of order I×L, since $\beta =\{\beta_{il}\}$ for i=1,..,I and l=1,..,L, $Z_{1}$ is of order n×J, $b_{1}$ is of order J×L and contains the genotype×trait interaction term since $b_{1}=\{gt_{jl}\}$ where $gt_{jl}$ is the effect of genotype×trait interaction term for j=1,..,J and for l=1,..,L.$\;Z_{2}$ is of order n×IJ, $b_{2}$ is of order IJ×L and contains the genotype×environment×trait interaction, since $b_{2}=\{gEt_{jil}\}$ where $gEt_{jil}$ is the effect of genotype×environment×trait interaction for j=1,..,J, for i=1,..,I and for l=1,..,L. Vector $b_{1}\;$is distributed under a matrix-variate normal distribution as $NM_{J\times L}\left(0,G_{g},\Sigma_{t}\right),$ where $G_{g}$ is of order J×J and represents the Genomic Relationship Matrix (GRM) and is calculated using the VanRaden (2008) method as $G_{g}=\frac{WW^{T}}{p}$, where p denotes the number of markers and W the matrix of markers of order J×p; and $\Sigma_{t}$ is the unstructured genetic (co)variance matrix of traits of order L×L, $b_{2}∼NM_{JI\times L}\left(0,\;\Sigma_{E}\;⊗G_{g},\Sigma_{t}\right)$, where $\Sigma_{E}\;$is an unstructured (co)variance matrix of order I×I and E is the matrix of residuals of order n×L with $E∼NM_{n\times L}\left(0,I_{n},R_{e}\right)$, where $R_{e}$ is the unstructured residual (co)variance matrix of traits of order L×L, and $G_{g}$ is the genomic relationship matrix described above (Montesinos-López *et al.*, 2018).

The BMTME model resulting from equation (1) was implemented by Montesinos-López *et al.* (2016). Next, we use the modified version of the Gibbs sampler of the original BMTME model proposed by Montesinos-López *et al.* (2016) that was implemented in Montesinos-López *et al.* (2018). It is important to point out that model (1) takes into account the genotype×environment terms in the ($Z_{2}b_{2})$ term and for comparison purposes, we also ran the model in equation (1) without the ($Z_{2}b_{2})$ term to study the effect on prediction performance with and without the genotype×environment term.

Outlined below is the Gibbs sampler implemented by Montesinos-López *et al.* (2018) for estimating the parameters of interest in the BMTME model. While the order is somewhat arbitrary, we suggest the following:

Step 1. Simulate β according to the normal distribution given in Appendix A (A.1) of Montesinos-López *et al.* (2018).
Step 2. Simulate $b_{1}\;$according to the normal distribution given in Appendix A (A.2) of Montesinos-López *et al.* (2018).
Step 3. Simulate $b_{2}\;$according to the normal distribution given in Appendix A (A.3) of Montesinos-López *et al.* (2018).
Step 4. Simulate $\Sigma_{t}\;$according to the inverse Wishart (IW) distribution given in Appendix A (A.4) of Montesinos-López *et al.* (2018).
Step 5. Simulate $\Sigma_{E}\;$according to the IW distribution given in Appendix A (A.5) of Montesinos-López *et al.* (2018).
Step 6. Simulate $R_{e}\;$according to the IW distribution given in Appendix A (A.6) of Montesinos-López *et al.* (2018).
Step 7. Return to step 1 or terminate when chain length is adequate to meet convergence diagnostics.

The main differences between this Gibbs sampler and that given by Montesinos-López *et al.* (2016) are: (i) the modified Gibbs sampler was built using the matrix-variate normal distribution instead of a multivariate normal distribution; (ii) this modified Gibbs sampler assumes a general or unstructured variance-covariance matrix for environments, that needs $\frac{L\times (L+1)}{2}$ parameters because every term is different, while the original BMTME model assumes a diagonal variance-covariance matrix for environments that only needs L parameters since all off diagonal elements are zero; and (iii) the original BMTME model used non-informative priors based on the Half-t distribution of each standard deviation term and uniform priors on each correlation of the covariance matrices of traits (genetic and residual). The priors implemented for the Gibbs sampler described above are given in Appendix A of this article.

#### Multi-trait deep learning (MTDL) model:

Popular neural network architectures are: (a) densely connected networks, (b) convolutional networks, and (c) recurrent networks. Details of each type of network, its assumptions and input characteristics can be found in Gulli and Sujit (2017), Angermueller *et al.* (2017) and Chollet and Allaire, (2017). In this study we implemented a type (a) network, which does not assume a specific structure in the input features. In general, the basic structure of a densely connected network consists of an input layer, L output layers (for multi-trait modeling) and multiple hidden layers between the input and output layers. This type of neural network is also known as a feedforward neural network. The implementation of this neural network is challenging because it requires the following hyperparameters: number of units (U), number of layers, number of epochs (E), type of regularization method and type of activation function. Based on the literature review, we decided to use the rectified linear activation unit (ReLU) as activation function and the dropout type of regularization method for training the models (Gulli and Sujit 2017; Angermueller *et al.*, 2017; and Chollet and Allaire 2017). The range of the remaining hyperparameters was determined by a few initial studies of a single fold (80% for training and 20% for testing), randomly selected from each data set; with these initial values, we implemented a full factorial design with 6 levels for the number of units and epochs and 3 levels for the number of layers. For more details on model selection in DL models, we suggest the companion paper of Montesinos-López *et al.* (2018), where the authors evaluate the prediction performance of univariate DL models for multi-environment data. In Appendix B we provide the R code for implementing MTDL models, while the R package BMTME that is still under development is available at the following link: https://github.com/frahik/BMTME.

### Experimental data sets

Three real data sets were analyzed, one data set comprising maize lines and two data sets comprising elite wheat lines. The three data sets included several environments.

#### Maize data set 1:

This data set was used by Crossa *et al.* (2013) and Montesinos-López *et al.* (2016) and is made up of a total of 309 maize lines. Three traits were evaluated: grain yield (GY), anthesis-silking interval (ASI), and plant height (PH); each of these traits was measured in three environments (Env1, Env2, and Env3) on the same 309 lines. Phenotypes of each trait were pre-analyzed and adjusted for the experimental field design. The genotyping was done with Genotype by Sequencing (GBS) technology with a total of 681,257 single nucleotide polymorphisms (SNPs); after filtering for missing values and minor allele frequency, we used 158,281 SNPs. Markers that had 80% of the maize lines with missing values were removed, and markers with a minor allele frequency lower than or equal to 0.05 were deleted.

#### Wheat data set 2:

This wheat data set is composed of 250 wheat lines that were extracted from a large set of 39 yield trials grown during the 2013-2014 crop season in Ciudad Obregon, Sonora, Mexico (Rutkoski *et al.*, 2016). The measured traits were plant height (PH) recorded in centimeters and days to heading (DTH) recorded as the number of days from germination until 50% of spikes had emerged in each plot, in the first replicate of each trial. Both traits were measured in three environments and on the same 250 lines. Phenotypes were also adjusted by experimental design. Genomic information was obtained by genotype by sequencing (GBS) and we used a total of 12,083 markers that remained after quality control. Single nucleotide polymorphism calls were extracted and markers were filtered so that percent missing data did not exceed 80%. Individuals with 80% missing marker data were removed, and markers were recorded as -1, 0, and 1, indicating homozygous for the minor allele, heterozygous, and homozygous for the major allele, respectively. Next, markers with 0.01 minor allele frequency were removed, and missing data were imputed with the marker mean.

#### Wheat Iranian data set 3:

This data set consists of 2374 wheat lines evaluated in a drought environment (D) and a heat environment (H) at the CIMMYT experiment station near Ciudad Obregon, Sonora, Mexico (27 ° 20 ′N, 109 ° 54 ′W, 38 meters above sea level) during the 2010-2011 cycle and was used in Crossa *et al.* (2016). Two traits were measured: days to maturity (DTM) and days to heading (DTH). Both traits were measured in the two environments and on the same 2374 lines. The number of markers used was 39,758 that remained after the quality control process from a total of 40,000 markers originally used.

### Experiments evaluated

In this empirical evaluation, we compared the prediction accuracy of the two proposed models: the MTDL method and the BMTME model. Both methods were implemented in the R statistical software (R Core Team 2018). The MTDL model was fitted with the Keras package (Gulli and Sujit 2017; Chollet and Allaire 2017) with a densely connected network architecture. In both MTDL and BMTME, we used two different sets of independent variables: the first set was composed of information on environments and genomes (that takes into account genomic information) ignoring genotype×environment interaction, while the second set included the genotype×environment term in addition to the main effects of environments and genomes. Under the MTDL model, we implemented a grid search for choosing the hyperparameters; this was done using a full factorial design with the following three factors: (a) number of units (U), (b) number of epochs (E), and (c) number of layers. These three factors are hyperparameters under the MTDL model. We studied the following values for the U and E hyperparameters: 50, 60, 70, 80, 90 and 100, and for the number of layers we used 1, 2 and 3. These values were chosen after the previous experiments were conducted. Thus 6×6×3 = 108 experiments were run for each data set with densely connected MTDL models.

It is important to point out that the 108 MTDL experiments used dropout regularization, which is one of the most effective and commonly used regularization techniques in neural networks; it was developed by Srivastava *et al.* (2014) at the University of Toronto. Dropout regularization is applied to a layer and consists of randomly dropping out (setting to zero) a number of the hidden layer’s during training. In our case, the dropout rate was 0.3 (30%); this meant that the percentage of features that were set to zero was 30% in each hidden layer (Gulli and Sujit 2017; Chollet and Allaire 2017).

#### Evaluation of prediction performance With cross-validation:

The prediction accuracy of both MTDL and BMTME models was evaluated with 10 random cross-validations (CV): the whole data set was divided into a training (TRN) and a testing (TST) set; 80% of the whole data set was assigned to TRN, and the remaining 20% was assigned to TST set. In our random CV, one observation can appear in more than one partition because we used sampling with replacement. However, the same observation is never included simultaneously in the training and testing sets of a random partition. In the design, some lines can be evaluated in some, but not all, target environments, which mimics a prediction problem faced by breeders in incomplete field trials. For this reason, our cross-validation strategy is exactly the same as that denoted by the CV2 proposed and implemented by Jarquín *et al.* (2017), where a certain portion of tested lines (genotypes) in a certain portion of tested environments are predicted since some tested lines that were evaluated in some tested environments are assumed missed in others. Since N=J×I denotes the total number of records per each available trait, then to select lines in the TST data set, we fixed the percentage of data to be used for TST (PTesting = 20%). Then 0.20×N (lines) were chosen at random, and subsequently one environment per line was randomly picked from I environments. The cells not selected through this algorithm were allocated to the TRN data set, while the selected cells (ij) were assigned to the TST data set. Lines were sampled with replacement if J<0.20×N, and without replacement otherwise (López-Cruz *et al.*, 2015). The metric used to measure the prediction accuracy of both models was the Pearson’s correlation and it was calculated from each trait-environment combination for each testing set of each random partition; thus the average of all partitions was reported as a measure of prediction performance. This explained cross validation method is called the outer CV and was applied for both models. However, in the DL model we also applied an inner CV strategy for tunning the hyperparameters using the grid of hyperparameter values defined above (108 experiments). The inner CV strategy consists in spliting each training set of the outer CV, here 20% of data were assigned for testing-inner and 80% for training-inner.The training-inner data set was used to train the DL model using the grid of hyperparameters values. This inner CV strategy was facilitated by using the internal capabilities of Keras by means of the validation_split argument on the fit() function. The predictve power is assessed in the second part of the data set (testing-inner). With this, a set of best-fitting hyperparameters (best combination of units, epoch and layers) from the inner CV loop is obtained. Finally, these set of hyperparameters were used to predict the performance in the independent testing data set (testing-outer). Since different traits in different environments have different heritabilities, we report the accuracy in terms of Pearson’s correlation divided by the square root of the heritability corresponding to each trait-environment combination.

### Data availability

The phenotypic and genotypic data used in this study can be downloaded from the following link: http://hdl.handle.net/11529/10548134.

## Results

Our results are given in four sections. In the three first sections we report the performance of each real data set analyzed, and in the last section we compare the prediction performance of both models for all data sets. In each of the first three sections, first we present a figure with prediction performance in terms of Pearson’s correlation under both models BMTME and MTDL with (I) and without (WI) the genotype×environment interaction term.

### Maize data set 1

**Figure 1:**
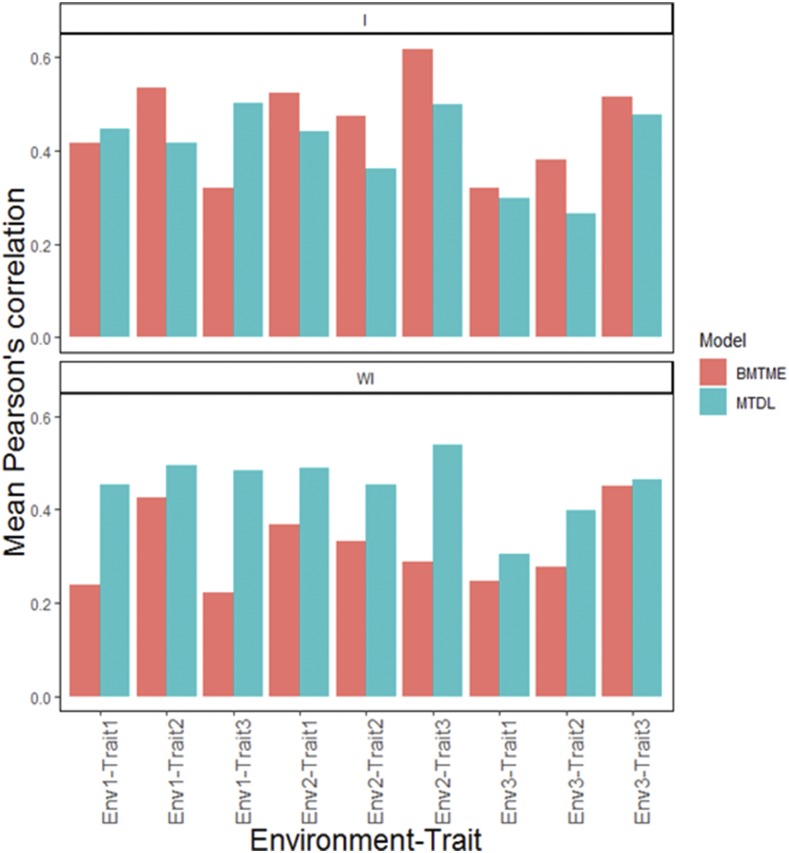
Maize data. Mean Pearson’s correlation for each environments-trait combination for the MTDL and GBLUP models. The top horizontal sub-panel corresponds to the model with genotype ×environment interaction (I), and the bottom horizontal sub-panel corresponds to the same model without genotype×environment interaction (WI).

Next we describe the prediction performance of the BMTME model without genotype× environment interaction (WI). The prediction accuracy of this model ranged from 0.222 to 0.451. Figure 1 gives the predictions under the MTDL model without the interaction term. Here Pearson’s correlation ranged from 0.305 to 0.540 (Figure 1). Next we present the prediction performance of the models including genotype ×environment interaction (I). First, under the BMTME model the predictions ranged from 0.321 to 0.619 in terms of Pearson’s correlation. The prediction performance of MTDL is shown in Figure 1; with the interaction term ranged from 0.266 to 0.501 in terms of Pearson’s correlation. The standard errors (SE) of the average Pearson correlation of this data set and of the following data sets are given in Table C1 of Appendix C.

### Wheat data set 2

**Figure 2:**
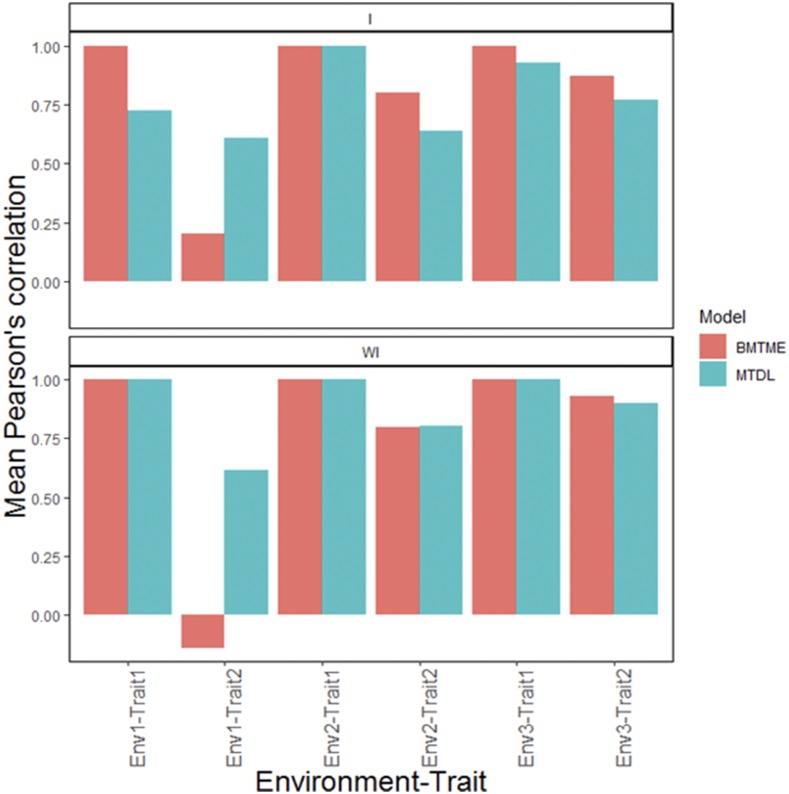
Wheat data. Means Pearson’s correlation for each environment-trait combination for the MTDL and GBLUP models. The top horizontal sub-panel corresponds to the model with genotype×environment interaction (I), and the bottom horizontal sub-panel corresponds to the same model without genotype×environment interaction (WI).

Next we describe the prediction performance of the BMTME model without genotype×environment interaction (WI). In terms of Pearson’s correlation, the range of predictions under this model was from -0.140 to 1.00 (Figure 2). Under the MTDL model without the interaction term, the predictions ranged from 0.613 to 1.00 and are given in Figure 2. When genotype×environment interaction (I) was considered under the BMTME model, the range of Pearson’s correlation was from 0.202 to 1.00 (Table C1). With the MTDL model the predictions ranged from 0.601 to 1.00 (Figure 2).

### Wheat Iranian data set 3

**Figure 3:**
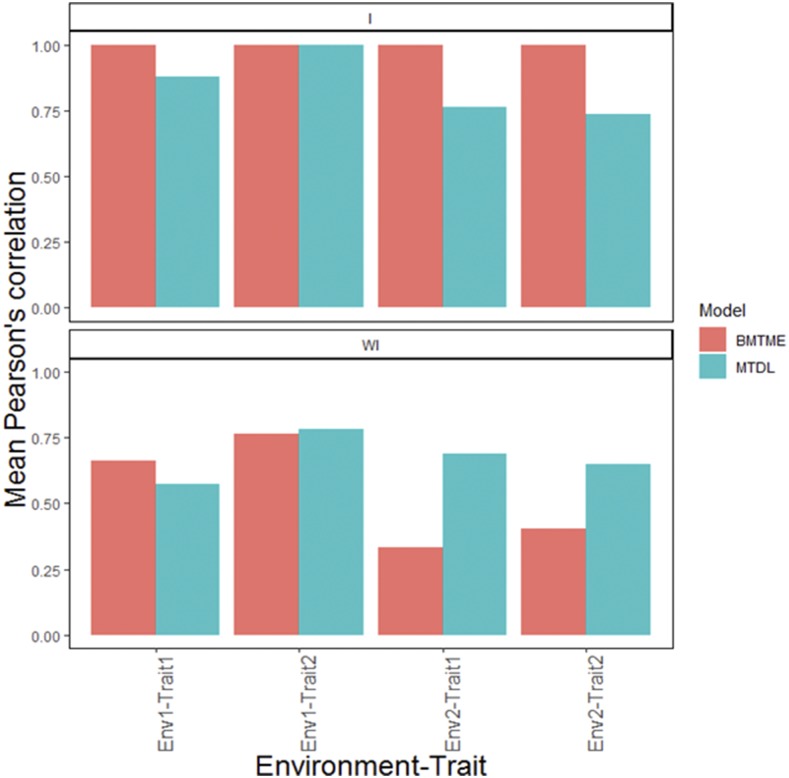
Iranian data. Mean Pearson’s correlation for each environments-trait combination for the MTDL and GBLUP models. The top horizontal sub-panel corresponds to the model with genotype×environment interaction (I), and the bottom horizontal sub-panel corresponds to the same model without genotype×environment interaction (WI).

The prediction performance of the BMTME model without genotype×environment interaction (WI) is presented first. The predictions of this model in terms of Pearson’s correlation ranged from 0.331 to 0.763 (Figure 3). Figure 3 gives also the predictions under the MTDL model without the interaction term for each environment-trait combination; the range of predictions with Pearson’s correlation was from 0.574 to 0.784. Next the predictions with genotype×environment interaction (I) are provided. First, under the BMTME model, the range of predictions in terms of Pearson’s correlation was from 0.998 to 1.00. The prediction performance of MTDL is shown in Figure 3, with the interaction term they ranged from 0.738 to 1.00.

### Comparing the BMTME model to the MTDL model

**Figure 4:**
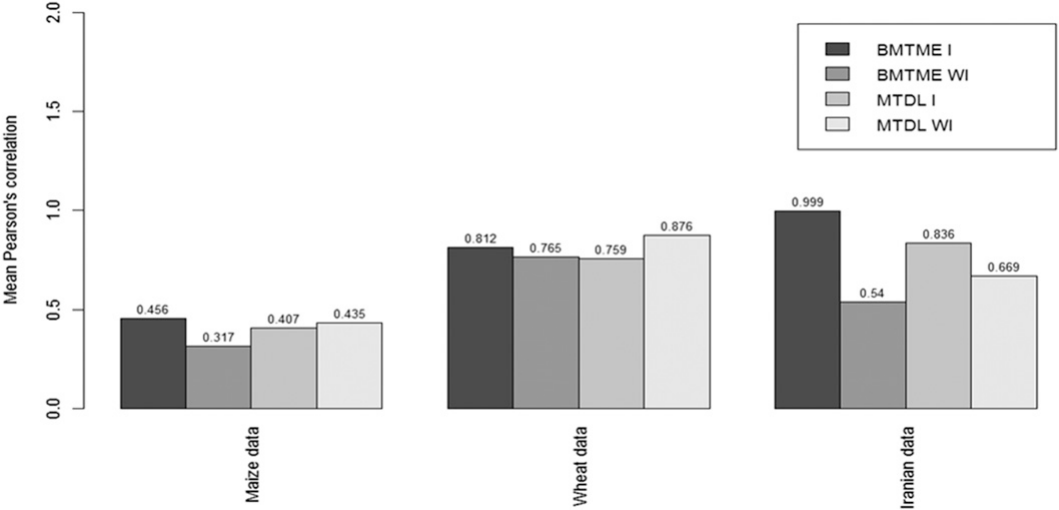
Mean Pearson’s correlation across environments-traits for the GBLUP and MTDL model conducted with (I) and without (WI) genotype×environment interaction for each data set.

To obtain a meta-picture of the prediction performance of the MTDL model against the BMTME model, we compared the average predictions across environments-traits of the MTDL model *vs.* those of the BMTME model for each data set. Figure 4 shows that when the genotype×environment interaction term was ignored in the three data sets under study, the best predictions were observed under the MTDL model, but when the genotype×environment interaction term was taken into account, the best predictions were obtained with the BMTME model. Finally, upon comparing the predictions for each data set with the four models resulting from the two models (BMTME and MTDL) and the two types of covariates used in the predictor (with and without the interaction term), we found that in maize data set 1, the best prediction of these four models corresponded to the BMTME model with the interaction term; this model was 4.61% better than the second best model, *i.e.*, MTDL without the interaction term. For wheat data set 2, the best prediction corresponded to the MTDL model without the genotype×environment interaction term; this model was 7.31% better than the second best model (BMTME with the interaction term). For the third data set, wheat Iranian data set 3, the best prediction corresponded to the BMTME model with the genotype×environment interaction term; this model was 16.32% better in terms of prediction accuracy than the second best model, MTDL with the interaction term.

## Discussion

The key objective of GS is to build an accurate prediction model based on training populations consisting of individuals with both genotypic and phenotypic data. For this reason, many univariate prediction models have been built and evaluated in the GS context. However, breeders usually select, at the same time, multiple traits that are often genetically correlated, with correlations that range from weak to strong. For this reason, in this paper we explored MTDL models for selecting candidate genotypes in GS that allow the simultaneous prediction of many traits measured in each experimental unit. We found evidence that MTDL models are very competitive in terms of prediction accuracy compared with BMTME models, since the prediction performance of MTDL models was competitive to that of the BMTME models. In general terms, the best predictions were produced by the BMTME model with the genotype×environment interaction term.

### Computation efficiency of MTDL vs. BMTME

Additionally, we found that the MTDL model is more computational efficiency than the BMTME model since for the maize and wheat data set, on average the MTDL was 11.71 and 3.39 times faster, respectively, than the BMTME model. For the maize data set the BMTME model required 42.222 hr for its implementation, whereas the MTDL only required 3.604 hr. For the wheat set BMTME required around 13.86 hr, while the MTME model only need 4.079 hr. However, it is important to point out that this difference can be due to the fact that the MTDL model automatically parallelized the jobs and its efficiency depends on the number of available cores. Furthermore, when comparing the BMTME model with the univariate Bayesian GBLUP model the BMTME model is considerable more demanding in terms of implementation time; however, we have not performed a formal comparison in this respect.

### Models Without genotype×environment interaction

For the three data sets, when genotype×environment interaction was ignored in both models, the best predictions were observed with the MTDL models compared to the BMTME model. This can be explained in part by the fact that DL models use hidden layers that automatically capture complex interaction terms without the need to pre-specify *a priori* covariates that include the interaction terms. This means that, unlike the BMTME model (and most statistical mixed models), DL models can capture not only two- or three-way interaction terms, but also interactions of larger order without the need to specify in the predictor the covariates corresponding to these interactions. However, the interpretability of DL models is not as transparent nor as easy as the interpretability of some genomic models such as the GBLUP and BMTME.

### Models With genotype×environment interaction

We found that in the three data sets when taking into account genotype×environment interaction in both models, the BMTME model was better than the MTDL model in terms of prediction accuracy. This means that although we found evidence that the MTDL model was very competitive in terms of prediction accuracy, the BMTME model was superior. Our results indicate that conventional prediction models based on mixed models (the BMTME model, in our case) are very powerful tools, with the advantage that they offer a transparent building process and interpretability, and avoid the time consuming and tedious task of tuning the hyperparameters that DL models need. However, we need to point out that our results are valid only for the hyperparameters used in this research.

### General comments

The performance of the MTDL model depends on the set of hyperparameters used; these hyperparameters are data dependent, which was corroborated in the three data sets used in this research. These different combinations of hyperparameters for each data set corroborate the difficulties found when choosing an appropriate set of hyperparameters in the implementation of MTDL models and of course we are aware that exploring other sets of hyperparameters the prediction of the MTDL model can be improved.

It is important to point out that the models (MTDL and BMTME) used in this research are only appropriate for multiple continuous traits; of course, each trait can be on a different scale. However, to successfully implement both models, we recommend rescaling the response variable for each training data set and, after getting the corresponding predictions for each testing set, transforming each variable back to its original scale. There are many ways to rescale the variables, but we used the standardization method (subtracting the mean and dividing by the standard deviation). However, other types of rescaling methods can be implemented and many times produce similar results in terms of prediction accuracy. The rescaling process is more important in DL models than in the BMTME model, since the BMTME works well even when the traits are in different scales.

One possible explanation for why multi-trait models often perform better than univariate models is that they effectively increase the sample size that is used to train the models. As different traits have different noise patterns, a model that learns two or more traits simultaneously is able to learn a more general representation. Learning just trait A bears the risk of overfitting to trait A, while learning A and B jointly enables the model to obtain a better representation by averaging the noise patterns.

Some explanations of why MTDL models are less efficient in terms of prediction accuracy than BMTME models are: (a) it is documented that DL in general requires a large amount of data for training to be successful, (b) MTDL models do not have a strong known theoretical foundation, (c) determining the architecture and the hyperparameters for DL is a challenging process and the way to proceed so far is just by trial and error, with no theory to guide the researcher, (d) lack of interpretation of results is indeed a problem, and (e) the system is prone to overfitting the training data. However, DL models also have some advantages: (a) they are easy to adapt to classification or numeric prediction problems, (b) they are capable of modeling more complex patterns than nearly any algorithm with less intense computer requirements, (c) they make few assumptions about the data’s underlying relationships, and (d) they are easy to adapt from univariate trait analysis to multi-trait analysis even with mixed phenotypes (binary, ordinal and continuous).

Finally, we would like to point out that our results are valid under a densely connected network architecture with the grid of hyperparameters used in the 108 experiments conducted. For this reason, we believe that with other types of network architecture and hyperparameters, it would be possible to achieve a better performance in terms of prediction accuracy of the MTDL models. For these reasons, we encourage conducting other studies to evaluate MTDL models in the context of genomic selection while exploring other network architectures and sets of hyperparameters.

## Conclusions

In this paper we propose using MTDL models for prediction in genomic selection. We found that the best predictions correspond to the BMTME model, but the predictions of the MTDL model were very competitive with those of the BMTME model, since in one of the three data sets, the prediction accuracy of the MTDL model was better than that of the BMTME model. We also found that the MTDL model performed better when the covariates corresponding to genotype×environment interaction were ignored. The implementation of the MTDL model also required less computational resources than the BMTME model. For these reasons, we have empirical evidence in favor of the MTDL model and suggest including these models in the toolkit of breeding scientists for predicting multiple traits simultaneously in GS. Although training the MTDL models is challenging due to the number of hyperparameters involved, we found that implementing MTDL models is feasible and practical in the GS context. However, more research is needed to increase the reliability of our findings.

## APPENDIX A

### Setting the hyperparameters for the prior distributions of the BMTME model

The hyperparameters for the BMTME model were set similar to those used in the BGLR software (Pérez and de los Campos, 2014). These rules provide proper but weakly informative prior distributions so that we partitioned the total variance-covariance of the phenotypes into two components: (1) the error and (2) the linear predictor. First we provide the variance-covariance of the phenotypes

$$Var(vec\left(Y\right))=\Sigma_{\beta t}⊗XX^{T}+\Sigma_{t}⊗Z_{1}G_{g}Z_{1}^{T}+\Sigma_{t}⊗Z_{2}(\Sigma_{E}⊗G_{g})Z_{2}^{T}+R_{e}⊗I_{n}$$ (B1)

Therefore, the variance-covariance of row i for i=1,2,…,n of Y is equal to

$$Var(y_{i})=\Sigma_{\beta t}x_{i}^{T}x_{i}+\Sigma_{t}z_{1i}^{T}G_{g}z_{1i}+\Sigma_{t}z_{2i}^{T}(\Sigma_{E}⊗G_{g})z_{2i}+R_{e}$$ (B2)

Therefore, the average of the n rows of equation (B2), called total variance, is equal to

$$Var\left({\bar{y}}_{i}\right)=\Sigma_{\beta t}\overset{n}{\underset{i=1}{\sum }}x_{i}^{T}x_{i}/n+\Sigma_{t}(\overset{n}{\underset{i=1}{\sum }}z_{1i}^{T}G_{g}z_{1i})/n+\Sigma_{t}(\overset{n}{\underset{i=1}{\sum }}z_{2i}^{T}(\Sigma_{E}⊗G_{g})z_{2i})/n+R_{e}$$

$$Var\left({\bar{y}}_{i}\right)=\Sigma_{\beta t}MS_{\beta t}+\Sigma_{t}MS_{b1}+\Sigma_{t}MS_{b2}+R_{e}$$

$$Var\left({\bar{y}}_{i}\right)=V_{y}=V_{\beta t}+V_{b1}+V_{b2}+R_{e}$$ (B3)

where: $MS_{\beta t}=\overset{n}{\underset{i=1}{\sum }}x_{i}^{T}x_{i}/n$, $MS_{b1}=(\overset{n}{\underset{i=1}{\sum }}z_{1i}^{T}G_{g}z_{1i})/n$, $MS_{b2}=(\overset{n}{\underset{i=1}{\sum }}z_{2i}^{T}(\Sigma_{E}⊗G_{g})z_{2i})/n$, and

$$V_{\beta t}=\Sigma_{\beta t}MS_{\beta t}$$ (B4)

$$V_{b1}=\Sigma_{t}MS_{b1}$$ (B5)

$$V_{b2}=\Sigma_{t}MS_{b2}$$ (B6)

#### Setting the hyperparameters for $\Sigma_{\mathrm{\beta t}}$:

Since $E(\Sigma_{\beta t}|df_{\beta t},S_{\beta t}$)=$\frac{S_{\beta t}}{df_{\beta t}-L-1}$ and mode$\left(\Sigma_{\beta t}|df_{\beta t},S_{\beta t}\right)=\frac{S_{\beta t}}{df_{\beta t}+L+1}$, for $df_{\beta t}>L+1$. Therefore, from equation (B4),

$$\Sigma_{\beta t}=V_{\beta t}/MS_{\beta t}$$ (B7)

Thus, if we replace the left-hand side of equation (B7) with the mode of $\Sigma_{\beta t}$, then

$$\frac{S_{\beta t}}{df_{\beta t}+L+1}=\frac{V_{\beta t}}{MS_{\beta t}}$$ (B8)

From (B8) and solving we get $S_{\beta t}=\frac{V_{\beta t}\times (df_{\beta t}+L+1)}{MS_{\beta t}}$. Then by setting $R_{1}^{2}$ as the proportion of the total variance-covariance ($V_{y}$) that is explained *a priori* by the traits, $V_{\beta t}=R_{1}^{2}V_{y}$, we have that

$$S_{\beta t}=\frac{R_{1}^{2}V_{y}\times (df_{\beta t}+L+1)}{MS_{\beta t}}$$ (B9)

Once we set $df_{\beta t}$, we can set $S_{\beta t}$ as in (B9) and we only need to compute the phenotypic variance-covariance matrix ($V_{y}$), $MS_{\beta t}$ and set $R_{1}^{2}$ as the proportion of variance-covariance that is explained *a priori* by the traits. We set as default $R_{1}^{2}=0.25$.

#### Setting the hyperparameters for $\Sigma_{t}$:

Also, since $E(\Sigma_{t}|df_{t1},S_{t1})=$$\frac{S_{t1}}{df_{t1}-L-1}$ and mode$\left(\Sigma_{t}|df_{t1},S_{t1}\right)=\frac{S_{t1}}{df_{t1}+L+1}$, for $df_{t1}>L+1$, and using equation (B5) and (B6), in a similar way as before

$$\Sigma_{t}=\frac{V_{b1}}{MS_{b1}}+\frac{V_{b2}}{MS_{b2}}$$ (B10)

Thus, if we replace the left-hand side of equation (B10) with the mode of $\Sigma_{t}$, then

$$\frac{S_{t}}{df_{t}+L+1}=\frac{V_{b1}}{MS_{b1}}+\frac{V_{b2}}{MS_{b2}}$$ (B11)

From (B11) and solving for $S_{t}=\frac{V_{b1}\times (df_{t}+L+1)}{MS_{b1}}+\frac{V_{b2}\times (df_{t}+L+1)}{MS_{b2}}$, then

$$S_{t}=\frac{R_{2}^{2}V_{y}\times (df_{t}+L+1)}{MS_{b1}}+\frac{R_{3}^{2}V_{y}\times (df_{t}+L+1)}{MS_{b2}}$$ (B12)

for which we only need to compute the phenotypic variance-covariance matrix ($V_{y}$), $MS_{b1},\;$ $MS_{b2}$ and set $R_{2}^{2}$ and $R_{3}^{2}$ as the proportion of variance-covariance that *a priori* is explained by the traits in the genotype×trait and genotype×environment×trait interaction terms. We set as default $R_{2}^{2}=R_{3}^{2}=0.25$. In the balanced case, $MS_{b2}=tr\left(Z_{2}\left(\Sigma_{\text{E}}⊗G\right)Z_{2}^{T}\right)=tr\left(Z_{2}^{T}Z_{2}\left(\Sigma_{\text{E}}⊗G\right)\right)=tr\left(\Sigma_{\text{E}}⊗G\right)=tr\left(G\right)tr\left(\Sigma_{\text{E}}\right)$, so to complete the setting value of $MS_{b2}$, we take $tr\left(\Sigma_{\text{E}}\right)=\frac{1}{tr\left(G\right)}\frac{1}{3L}\overset{L}{\underset{l=1}{\sum }}V_{y_{l}}$, where $V_{y_{l}}$ is the phenotypic variance of trait l.

#### Setting the hyperparameters for $\Sigma_{E}$:

Also, since $E(\Sigma_{E}|df_{E},S_{E})=$$\frac{S_{E}}{df_{E}-I-1}$ and mode$\left(\Sigma_{E}|df_{E},S_{E}\right)=\frac{S_{E}}{df_{E}+I+1}$, for $df_{E}>I+1$. Let$\;V_{y*}$ be the variance-covariance matrix of the matrix of phenotypic responses, $Y^{*}$, that resulted from accommodating the information of the matrix of phenotypic responses (Y) of order n×L, with n = IJ, as a matrix of order $n^{*}\times I$, with $n^{*}=JL$, that is, the columns of $Y^{*}$ correspond to environments instead of traits as in Y. Then, in similar fashion, we can define

$$\Sigma_{E}=V_{b2*}/MS_{b2*}$$ (B13)

Thus, if we replace the left-hand side of equation (B13) with the mode of $\Sigma_{E}$, then

$$\frac{S_{E}}{df_{E}+L+1}=\frac{V_{b2*}}{MS_{b2*}}$$ (B14)

from (B14) and solving for $S_{E}=\frac{V_{b2*}\times (df_{E}+L+1)}{MS_{b2}}$, then

$$S_{E}=\frac{R_{3}^{2}V_{y*}\times (df_{E}+I+1)}{MS_{b2*}}$$ (B15)

with $MS_{b2*}=tr\left(\frac{S_{t}}{df_{t}+L+1}⊗G\right)$

#### Setting the hyperparameters for $R_{e}$:

Also, since $E(R_{e}|df_{e},S_{e}$)=$\frac{S_{e}}{df_{e}-L-1}$ and mode$\left(R_{e}|df_{e},S_{e}\right)=\frac{S_{e}}{df_{e}+L+1}$, for $df_{e}>L+1$. Therefore, in similar fashion to the above hyperparameters, we set

$$S_{e}=(1-R_{1}^{2}-R_{2}^{2}-R_{3}^{2})V_{y}\times (df_{e}+L+1)$$ (B16)

## APPENDIX B

### R code for implementing MTDL models

setwd(“C:/TELEMATICA 2017/Deep Learning Multi-trait”)

rm(list = ls())

######Libraries required#################################

library(tensorflow)

library(keras)

#############Loading data################################

load(“Data_Maize_set_1.RData”)

ls()

####Genomic relationship matrix (GRM)) and phenotipic data######

G=G_maize_1to3

Pheno=Pheno_maize_1to3

head(Pheno)

###########Cholesky decomposition of the GRM######

LG=t(chol(G))

########Creating the desing matrices #################

Z1=model.matrix(∼0+as.factor(Pheno$Line))

ZE=model.matrix(∼0+as.factor(Pheno$Env))

Z1G=Z1%*%LG

Z2GE=model.matrix(∼0+as.factor(Pheno$Line):as.factor(Pheno$Env))

G=data.matrix(G)

G2=kronecker(diag(3),G)

LG2=t(chol(G2))

Z2GE=Z2GE%*%LG2

###Defining the number of epoch and units#####################

units_M=50

epochs_M=50

##########Data for trait GY#################################

y = Pheno[,3:5]

X = cbind(ZE,Z1G,Z2GE)

head(y)

#############Training and testing sets###################

n=dim(X)[1]

nt=dim(y)[2]

Post_trn=sample(1:n,round(n*0.8))

X_tr = X[Post_trn,]

X_ts = X[-Post_trn,]

y_tr = scale(y[Post_trn,])

Mean_trn=apply(y[Post_trn,],2,mean)

SD_trn=apply(y[Post_trn,],2,sd)

y_ts=matrix(NA,ncol=nt,nrow=dim(X_ts)[1])

for (t in 1:nt){

y_ts[,t] =(y[-Post_trn,t]- Mean_trn[t])/SD_trn[t] }

# add covariates (independent variables)

input <- layer_input(shape=dim(X_tr)[2],name=“covars”)

# add hidden layers

base_model <- input %>%

layer_dense(units =units_M, activation=’relu’) %>%

layer_dropout(rate = 0.3) %>%

layer_dense(units = units_M, activation = “relu”) %>%

layer_dropout(rate = 0.3) %>%

layer_dense(units = units_M, activation = “relu”) %>%

layer_dropout(rate = 0.3)

# add output 1

yhat1 <- base_model %>%

layer_dense(units = 1, name=“yhat1”)

# add output 2

yhat2 <- base_model %>%

layer_dense(units = 1, name=“yhat2”)

# add output 3

yhat3 <- base_model %>%

layer_dense(units = 1, name=“yhat3”)

# build multi-output model

model <- keras_model(input,list(yhat1,yhat2,yhat3)) %>%

compile(optimizer = “rmsprop”,

loss=”mse”,

metrics=”mae”,

loss_weights=c(0.3333,0.3333,0.3333))

# fit model

model_fit <- model %>%

fit(x=X_tr,

y=list(y_tr[,1],y_tr[,2],y_tr[,3]),

epochs=epochs_M,

batch_size = 50,

verbose=0)

# predict values for test set

Yhat <- predict(model, X_ts) %>%

data.frame() %>%

setNames(colnames(y_tr))

predB=Yhat

y_p=predB

for (s in 1:nt){

y_p[,s]=y_p[,s]*SD_trn[s]+ Mean_trn[s]

y_ts[,s]=y_ts[,s]*SD_trn[s]+ Mean_trn[s]

}

#################Observed and predicted values############

Y_all_tst = data.frame(cbind(y_ts, y_p))

Y_all_tst

########Prediciton accuracy with Pearson Correlation######

Cor_Mat=cor(Y_all_tst)

Cor_Traits=diag(Cor_Mat[(nt+1):(2*nt),1:nt])

Cor_Traits

########Plots of observed and predicted values############

plot(Y_all_tst[,1],Y_all_tst[,4])

plot(Y_all_tst[,2],Y_all_tst[,5])

plot(Y_all_tst[,3],Y_all_tst[,6])

## APPENDIX C

**Table C1.** Standard errors (SE) of both models, BMTME and MTDL for each data set. WI denotes the scenarios that ignore the genotype×environment interaction term, while I denotes the scenarios with the genotype×environment interaction term. Env_Trait denotes the environment-trait combination

		Maize		Wheat		Iranian Wheat	
Type	Env_Trait	BMTME	MTDL	BMTME	MTDL	BMTME	MTDL
WI	Env1_Trait_1	0.042	0.046	0.022	0.031	0.014	0.025
WI	Env1_Trait_2	0.047	0.030	0.052	0.061	0.018	0.033
WI	Env1_Trait_3	0.051	0.048	—	—	—	—
WI	Env2_Trait_1	0.045	0.048	0.013	0.020	0.018	0.029
WI	Env2_Trait_2	0.032	0.055	0.032	0.041	0.028	0.030
WI	Env2_Trait_3	0.036	0.052	—	—	—	—
WI	Env3_Trait_1	0.030	0.058	0.018	0.027	—	—
WI	Env3_Trait_2	0.046	0.053	0.027	0.039	—	—
WI	Env3_Trait_3	0.054	0.044	—	—	—	—
I	Env1_Trait_1	0.027	0.046	0.021	0.041	0.008	0.025
I	Env1_Trait_2	0.029	0.029	0.059	0.054	0.006	0.030
I	Env1_Trait_3	0.026	0.036	—	—	—	—
I	Env2_Trait_1	0.032	0.042	0.019	0.023	0.013	0.030
I	Env2_Trait_2	0.035	0.062	0.025	0.041	0.007	0.030
I	Env2_Trait_3	0.038	0.042	—	—	—	—
I	Env3_Trait_1	0.026	0.049	0.018	0.038	—	—
I	Env3_Trait_2	0.041	0.051	0.020	0.045	—	—
I	Env3_Trait_3	0.040	0.034	—	—	—	—
